# ChatGPT-4o and OpenAI-o1: A Comparative Analysis of Its Accuracy in Refractive Surgery

**DOI:** 10.3390/jcm14155175

**Published:** 2025-07-22

**Authors:** Avi Wallerstein, Taanvee Ramnawaz, Mathieu Gauvin

**Affiliations:** 1 Department of Ophthalmology and Visual Sciences, McGill University, Montreal, QC H4A 0A4, Canada; mgauvin@lasikmd.com; 2 LASIK MD, Montreal, QC H3B 4W8, Canada; 3 School of Medicine, University of Montreal, Montreal, QC H3T 1J4, Canada; taanvee.punam.ramnawaz@umontreal.ca

**Keywords:** artificial intelligence, large language models, ChatGPT, refractive surgery, ophthalmology

## Abstract

**Background:** To assess the accuracy of ChatGPT-4o and OpenAI-o1 in answering refractive surgery questions from the AAO BCSC Self-Assessment Program and to evaluate whether their performance could meaningfully support clinical decision making, we compared the models with 1983 ophthalmology residents and clinicians. **Methods**: A randomized, questionnaire-based study was conducted with 228 text-only questions from the Refractive Surgery section of the BCSC Self-Assessment Program. Each model received the prompt, “Please provide an answer to the following questions.” Accuracy was measured as the proportion of correct answers and reported with 95 percent confidence intervals. Differences between groups were assessed with the chi-squared test for independence and pairwise comparisons. **Results**: OpenAI-o1 achieved the highest score (91.2%, 95% CI 87.6–95.0%), followed by ChatGPT-4o (86.4%, 95% CI 81.9–90.9%) and the average score from 1983 users of the Refractive Surgery section of the BCSC Self-Assessment Program (77%, 95% CI 75.2–78.8%). Both language models significantly outperformed human users. The five-point margin of OpenAI-o1 over ChatGPT-4o did not reach statistical significance (*p* = 0.1045) but could represent one additional correct decision in twenty clinically relevant scenarios. **Conclusions**: Both ChatGPT-4o and OpenAI-o1 significantly outperformed BCSC Program users, demonstrating a level of accuracy that could augment medical decision making. Although OpenAI-o1 scored higher than ChatGPT-4o, the difference did not reach statistical significance. These findings indicate that the “advanced reasoning” architecture of OpenAI-o1 offers only incremental gains and underscores the need for prospective studies linking LLM recommendations to concrete clinical outcomes before routine deployment in refractive-surgery practice.

## 1. Introduction

The rapid advancement of artificial intelligence has brought large language models (LLMs) to the forefront of clinical research and medical decision support. These models are being integrated into healthcare workflows due to their ability to synthesize complex information and provide on-demand guidance in time-sensitive environments. LLMs are already being widely used as ancillary tools in domains such as medical imaging analysis, patient triaging, and research support. In ophthalmology, recent studies have evaluated large language models across various areas of ophthalmology using standardized question banks such as the AAO BCSC, OphthoQuestions, and FRCOphth exam materials, StatPearls, as well as author-curated question banks. While ChatGPT-4 has consistently outperformed earlier versions like ChatGPT-3.5 and other models such as Bing AI and Google Bard (now called Gemini), these evaluations have focused on general ophthalmology [1,2,3,4,5,6,7,8,9,10,11] or select subspecialties like age-related macular degeneration [12], myopia care [13], choroidal melanoma [14], or retinopathy of prematurity [15]. The reliability of LLMs in the highly specialized subdiscipline of refractive surgery has limited investigation. Yet refractive surgeries are performed on millions of eyes each year, ranging from otherwise healthy corneas seeking refractive freedom to pathologic cases such as cataracts, post-LASIK ectasia, or keratoconus that require visual rehabilitation. Any decision support tool that sharpens preoperative planning can directly lower complication rates, reduce postoperative refractive surprises, and enhance patient satisfaction and outcomes.

Refractive surgery is a uniquely demanding subspecialty that blends optical physics, corneal biomechanics, and patient-specific visual goals. Clinical decision making requires integrating biometric data, topographic interpretation, risk stratification for postoperative complications, and a nuanced understanding of evolving surgical technologies. These include corneal procedures such as LASIK, PRK, and SMILE, as well as intraocular procedures like phakic intraocular lenses (PIOLs), refractive lens exchange, and cataract surgery with premium intraocular lens (IOL) implantation. Surgeons must also weigh patient-specific factors such as pupil size, corneal irregularity and dry eye risk, while counseling patients on visual quality expectations, glare, halos, and long-term stability. Because many refractive interventions (e.g., LASIK, PIOLs, refractive lens exchange) are elective and expectation-sensitive, whereas cataract surgery is medically indicated yet still outcome-sensitive, even modest improvements in planning or counseling translate into tangible clinical value for any patient groups. These multifactorial considerations make refractive surgery an ideal domain to assess whether large language models can simulate real-world ophthalmic reasoning sufficiently well to support clinicians at the point of care.

Our group recently conducted the first study evaluating LLM performance in the subspecialty of refractive surgery, using 100 standardized questions from the AAO BCSC Self-Assessment Program [16]. Five widely used models were compared, namely ChatGPT-4, ChatGPT-4o, Gemini, Gemini Advanced, and Copilot. ChatGPT-4o consistently outperformed all other models, including its predecessor, and was the only LLM to statistically surpass the average performance of ophthalmology residents. On 5 December 2024, OpenAI released OpenAI-o1, a new model claiming enhanced “advanced reasoning” capabilities over previous iterations, including ChatGPT-4o [17]. Whether those upgrades cross a threshold at which model guidance could safely inform surgical planning, however, remains unknown.

Building on our prior findings [16], the present study investigates whether OpenAI-o1 represents a measurable advancement over ChatGPT-4o in the subspecialty of refractive surgery, a complex and data-intensive domain within ophthalmology. Using a larger dataset of 228 questions from the Refractive Surgery section of the AAO BCSC Self-Assessment Program, we directly compared the performance of both models and benchmarked their accuracy against the scores of 1983 ophthalmology residents and clinicians who previously completed the same questions on the platform. Our primary aim was to determine whether any observed performance gain is clinically meaningful, defined as a statistically and practically significant margin over resident performance that could justify integrating the model as a decision support aid during patient work-up and surgical counseling. This study not only evaluates whether recent architectural improvements in OpenAI-o1 translate into statistically significant gains over its predecessor but also contributes to defining the role of LLMs as reliable tools for subspecialty-level clinical decision support with clear, actionable implications for real-world refractive-surgery practice.

## 2. Materials and Methods

### 2.1. Compared Large Language Models

The study assessed the accuracy of leading AI chatbots, namely ChatGPT-4o and OpenAI-o1 (OpenAI, San Francisco, CA, USA). While GPT-4o offers improvements in its textual, vocal, pictorial and linguistic analyses, GPT-o1 distinguishes itself from its former versions by allocating more processing time and using “longer chains of thought” to enhance its precision and accuracy [18,19,20,21].

### 2.2. Question Selection

The BCSC Self-Assessment Program, offered by the American Academy of Ophthalmology (San Francisco, CA, USA), is an online learning platform consisting of 4522 questions across 13 ophthalmology subspecialities. Amongst these, the refractive surgery section contains 256 questions. For this study, and with permission from the American Academy of Ophthalmology, questions requiring image interpretation were excluded, generating 228 text-only questions. The multiple-choice questions all provided four options labeled either A, B, C, or D from which test-takers select the single correct answer. The approval from the Research Ethics Board was not required, as the study did not involve human or animal participants and the data collection did not contain any form of personal information.

### 2.3. Prompt Methodology and Data Entry

Our previous study entitled “Evaluating Large Language Models vs. Residents in Cataract & Refractive Surgery: A Comparative Analysis Using the AAO BCSC Self-Assessment Program” concluded that contextualized prompts asking for best-answer selection did not significantly improve language model performance [16]. Thus, the current study utilized the following prompt: “*Please provide an answer to the following questions*.” As such, one session was conducted for each of the models in which such instruction headed the questionnaire. In addition, a privacy request was previously submitted for the account used in this study to prevent data retention or cross-contamination between trials. Hence, no input was used for further training, and therefore the first session conducted using ChatGPT-4o did not impact the following session conducted on OpenAI-o1. All prompts were submitted to the models in a fresh session to reduce memory-based confounding. This design allowed us to simulate a realistic interaction with each model under controlled conditions, comparable to a single-use clinical query. We acknowledge that LLM outputs can vary slightly between runs due to the stochasticity inherent in model generation. Although formal repeatability testing was not conducted in this study, our prompt standardization and privacy isolation protocols were intended to minimize variability and simulate real-world single-query use.

### 2.4. Response Assessment and Recording

Similarly to our previous study, the models’ selected answers were recorded, and if the response did not include one of the provided options (A, B, C, or D), the chatbot’s response was automatically recorded as being incorrect. This approach simulated the examination setting, where multiple-choice questions left unanswered were considered incorrect.

### 2.5. BCSC Self-Assessment User Statistics Regarding the Refractive Surgery Section

Obtained with the help of American Academy of Ophthalmology, a report averaged the scores for the questions pertaining to the Refractive Surgery section of all users of the BCSC Self-Assessment Program in the past 12 months, which eventuated to the usage of the data for 1983 users.

### 2.6. Data and Statistical Analysis

Performance was measured by calculating the proportion of correct answers provided by each model. To ensure a robust comparison between models, performance evaluation was not limited to raw accuracy. We applied statistics to determine whether observed differences were statistically meaningful. To determine if one of the models performed in a manner significantly different to the others, a chi-squared test to assess for independence was conducted. A 95% confidence interval was reported for each estimate and statistical significance was set to a *p*-value of less than 0.05.

## 3. Results

A total of 228 questions, selected to exclude those requiring image interpretation, were included in the analysis. ChatGPT-4o answered 197 questions correctly, corresponding to 86.4% accuracy (95% CI, 81.9–90.9%). OpenAI-o1 answered 208 questions correctly, achieving 91.2% accuracy (95% CI, 87.6–95.0%) (Table 1). When compared to the average score of 1983 BCSC users (77%, 95% CI, 75.2–78.8%), both ChatGPT-4o and OpenAI-o1 demonstrated statistically superior performance.

**Figure 1:**
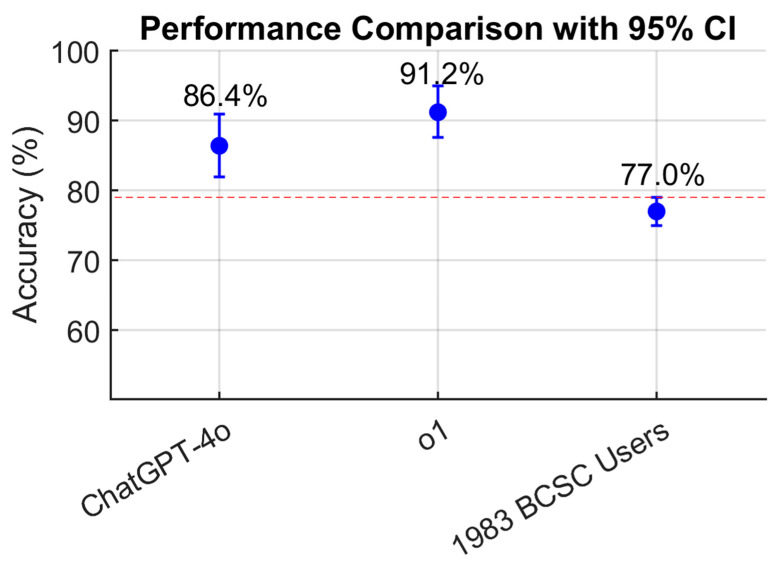
Comparison of ChatGPT-4o, OpenAI-o1, and resident performance displayed as a dot plot with 95% confidence intervals, illustrating the higher accuracy of both models relative to the resident average. The red dashed line indicates the value of the upper CI of the residents’ score (79%).

As illustrated in Figure 1, a dot plot with vertical error bars demonstrates that the CIs for ChatGPT models do not include the 95% CI margins of the BCSC users’ performance (red dashed line), indicating a greater and statistically superior performance relative to the resident benchmark (*p* < 0.05). Markedly, OpenAI-o1 obtained a 14.2% higher score than the average BCSC user grade (*p* < 0.05). However, while OpenAI-o1 outperformed, obtaining a mark 4.8% higher than ChatGPT-4o, this difference was not statistically significant (*p* = 0.1045).

## 4. Discussion

This study evaluated the accuracy of two current LLMs, ChatGPT-4o and OpenAI-o1, when tested on refractive-surgery questions from the AAO BCSC Self-Assessment Program, an online platform often used by ophthalmology residents to prepare for their Ophthalmic Knowledge Assessment Program (OKAP) examination. The questions were obtained with the assistance of the AAO. By benchmarking the models against a real-world cohort of 1983 residents and practicing clinicians, our evaluation is rooted in evidence that directly impacts clinical medicine rather than relying on abstract AI-only comparisons.

OpenAI-o1 (91.2%, 95% CI 87.6–95.0%) obtained a higher score than ChatGPT-4o (86.4%, 95% CI 81.9–90.9%), although the difference did not reach statistical significance (*p* = 0.1045). However, both LLMs significantly outperformed the users of the BCSC Self-Assessment Program. This 4.8% difference, although not statistically significant, is still noteworthy. A five-percentage-point margin corresponds to one extra correct surgical decision in twenty, which could mean identifying a subtle topographic contraindicator to LASIK, selecting a more appropriate phakic IOL size or recognizing an early ectasia pattern that warrants cross-linking rather than surgery. Similarly to findings in other domains, architectural refinements in newer LLMs may yield only marginal gains in narrowly scoped, high-performing domains such as refractive surgery. As models approach ceiling-level performance, additional improvements tend to produce diminishing returns. This highlights the importance of critically evaluating marketing claims about “advanced reasoning” by validating model performance against rigorous task-specific benchmarks.

Notably, ChatGPT-4o behaved in a manner similar to its performance in our previous publication comparing five different LLMs. In our previous study, ChatGPT-4o was given 100 questions from the Refractive Surgery section of the BCSC Self-Assessment Program [16], achieving accuracies of 84% (95% CI 77–91%) with a simple prompt and 86% (95% CI 79–93%) with a contextualized prompt [16]. Despite the larger sample size of 228 questions in the current study, ChatGPT-4o’s accuracy remained comparable, showing how reproducible its ability is. Such consistency is clinically relevant, because decision support tools must deliver stable recommendations across encounters and institutions.

It is also important to underscore that, unlike the human BCSC users, who typically train using the official BCSC manuals and other curated course materials, the LLMs evaluated in this study had no such targeted preparation for the material. Most high-quality ophthalmology content, including the BCSC series, is proprietary and was not part of the training corpus made available to OpenAI’s models. While LLMs are trained on a wide array of open access content, they cannot retrieve pay-walled academic content. Thus, the fact that ChatGPT-4o and OpenAI-o1 achieved higher accuracy than human users without direct exposure to the source material highlights the remarkable generalization ability of these models. For clinicians, this implies that LLMs could already function as real-time “second readers” during pre-operative workups, even without bespoke fine-tuning, thereby reducing cognitive load and standardizing care. It is likely that if these models had been fine-tuned on BCSC specific content, their performance would likely have been even higher. While raw accuracy metrics, such as the percentage of accurately answered questions, provide a quantitative basis for comparison, they do not fully capture the reliability or clinical applicability of LLMs in educational or decision support contexts. For instance, an LLM might answer correctly by chance or without reliable internal reasoning, and accuracy scoring alone may mask issues like overconfidence in incorrect responses or inconsistency across content domains. Future work should pair accuracy with calibration analyses, session-to-session reproducibility, and prospective simulations that track downstream outcomes such as complications rates or patient-reported quality of vision and satisfaction.

### Limitations

This study demonstrates that both ChatGPT-4o and OpenAI-o1 significantly outperformed the users of the BSCS Self-Assessment program. However, while OpenAI-o1 obtained a higher score than ChatGPT-4o, this difference was not statistically significant. This may partially be explained by the relatively small sample size of 228 questions. If the database had a larger number of questions, the observed difference could have achieved statistical significance with an estimated *p*-value smaller than 0.05.

Another limitation lies in the diversity of the user base of the BCSC comparison group, which included individuals ranging from first-year ophthalmology residents to experienced ophthalmologists in practice. The exact proportion of refractive-surgery subspecialists among the 1983 BCSC users is unknown, and it is likely that a cohort composed exclusively of refractive experts would have achieved a higher average score than the observed 77%. Nonetheless, the fact that LLMs reached a 92% score in this context remains noteworthy and highlights their potential as clinical decision support tools, even when benchmarked against a broad real-world user base. Additionally, the self-assessment platform enables users to answer questions multiple times, with their scores reflecting their most recent attempt rather than a first-time result. This could lead to inflated scores that do not accurately represent first-time knowledge or decision making capabilities.

Finally, the rapid release cycle of LLMs represents an ongoing challenge for longitudinal validation. Since completing data collection, OpenAI has already introduced newer iterations beyond o1, including o3 and o4-mini. These evolving versions can exhibit fluctuating performance characteristics, making it critical to treat each evaluation as a time-stamped benchmark rather than a definitive ranking. Adopting version-controlled benchmarking, coupled with prospective clinical trials that integrate LLM suggestions into refractive-surgery planning and measuring hard endpoints such as enhancement frequency or uncorrected visual acuity, will be essential to determine whether these tools translate into measurable patient benefit.

## 5. Conclusions

In this focused evaluation of two current large language models, ChatGPT-4o and OpenAI-o1 both outperformed 1983 ophthalmology residents and clinicians on 228 refractive-surgery questions from the AAO BCSC Self-Assessment Program, a validated benchmark that mirrors routine clinical decision making in ophthalmology and medicine. Both models demonstrated excellent generalization abilities within a highly specialized domain, without task-specific fine-tuning. OpenAI-o1 achieved the highest accuracy, of 91.2 percent versus 86.4 percent for ChatGPT-4o, yet this margin did not reach statistical significance in this current sample, although even a five-point edge could translate into fewer missed ectasia risks or suboptimal IOL choices in real patients. These findings suggest that while Open AI’s newer “advanced reasoning” architecture shows promise, its incremental gains must be weighed against real-world workflow integration and safety considerations. Future validation should couple version-controlled benchmarking with prospective clinical studies that measure tangible endpoints such as complications rates, uncorrected and corrected visual acuity, and patient satisfaction. Continuous evaluation will be critical to assess model improvements over time before LLMs can be adopted safely as an educational or medical decision-support tool that directly affects clinical outcomes in refractive-surgery practice.

## Figures and Tables

**Table 1:** A detailed summary of the correct responses, total questions, and corresponding 95% confidence intervals for both models and the resident cohort.

LLM	Questions Correct	Total Questions	Accuracy (%)	95% CI
ChatGPT-4o	197	228	86.4	81.9–90.9
OpenAI-o1	208	228	91.2	87.6–95.0
1983 BCSC Users	Not applicable	Not applicable	77.0	~75–79

## Data Availability

The original contributions presented in this study are included in the article. Further inquiries can be directed to the corresponding author.
